# Effects of alpha-lipoic acid on sperm quality in patients with varicocele-related male infertility: study protocol for a randomized controlled clinical trial

**DOI:** 10.1186/s13063-022-06951-0

**Published:** 2022-12-12

**Authors:** Qi-Feng Zhang, Sheng Wang, Han Zhang, Qi-Li Liu, Yu Wei, Wei Deng, Chuang Wang, Bo Yang

**Affiliations:** 1 Department of Andrology, Guilin People’s Hospital, Guilin, 541002 China; 2 Department of Urology, Guilin People’s Hospital, Guilin, 541002 China; 3 Department of Vascular Surgery, Affiliated Hospital of Guilin Medical University, Guilin, 541002 China

**Keywords:** Alpha-lipoic acid, Sperm quality, Varicocele, Male infertility, Varicocele-related male infertility

## Abstract

**Background:**

Varicocele is a high incidence and is considered to be the most common and correctable cause of male infertility. Oxidative stress (OS) plays a central role in the pathogenesis of varicocele-related male infertility. In addition to varicocelectomy, antioxidant supplementation seems to be an effective scheme for the treatment of varicocele-related male infertility, but it is still controversial. The purpose of this study is to determine the effects of alpha-lipoic acid (ALA) supplementation on sperm quality in patients with varicocele-related male infertility.

**Methods:**

In this randomized controlled clinical trial, we will randomize 80 patients with varicocele-related male infertility from Guilin People’s Hospital. The non-surgical observation group (*n* = 20) will receive ALA, the non-surgical control group (*n* = 20) will receive vitamin E, the surgical observation group (*n* = 20) will receive ALA after the operation, and the surgical control group (*n* = 20) will receive vitamin E after the operation. The course of treatment will be 3 months. The results will compare the changes in semen parameters, sex hormones, testicular volume, sperm DNA fragment index (DFI), seminal plasma malondialdehyde (MDA), and total antioxidant capacity (TAC) between the groups at baseline and after 3 months of antioxidant supplementation.

**Discussion:**

Whether it is necessary to use antioxidants in varicocele-related male infertility, how potent antioxidants should be used, postoperative application or non-surgical independent application still needs to be explored. This study attempts to compare the effects of two antioxidants (ALA and vitamin E) on sperm quality in patients with varicocele-related male infertility (surgical or non-surgical) and attempted to answer the above questions.

**Trial registration:**

Chinese Clinical Trial Registry (ChiCTR) ChiCTR2100054958. Registered on 29 December 2021

## Administrative information

Note: The numbers in curly brackets in this protocol refer to the SPIRIT checklist item numbers. The order of the items has been modified to group similar items (see http://www.equator-network.org/reporting-guidelines/spirit-2013-statement-defining-standard-protocol-items-for-clinical-trials/).

**Table:** 

Title{1}	Effects of alpha-lipoic acid on sperm quality in patients with varicocele-related male infertility: study protocol for a randomized controlled clinical trial
Trial registration {2a and 2b}.	ChiCTR.org.cn, ID: ChiCTR2100054958. Prospectively registered on 29 December 2021.
Protocol version {3}	December 29, 2021, version 1.
Funding {4}	No external funding.
Author details {5a}	Qi-Feng Zhang^1^, Sheng Wang^2^, Han Zhang^2^, Qi-Li Liu^3^, Yu Wei^2^, Wei Deng^2^, Chuang Wang^2^ and Bo Yang^2^ 1. Department of Andrology, Guilin People’s Hospital, Guilin 541002, China 2. Department of Urology, Guilin People’s Hospital, Guilin 541002, China 3. Department of Vascular Surgery, Affiliated Hospital of Guilin Medical University, Guilin 541002, China
Name and contact information for the trial sponsor {5b}	Guilin People’s Hospital Address: 12# Wenming Road, Guilin, 541002, China Tel: +86 0773 2823931
Role of sponsor {5c}	This is a researcher-driven study carried out in Guilin People’s Hospital, with no outside sponsor or funding.

## Introduction

### Background and rationale {6a}

It is estimated that about 8–12% of couples suffer from infertility, nearly half of which are mainly caused by male factors. The causes of male subfertility can be related to congenital, acquired, or idiopathic factors that impair spermatogenesis [1]. Among the acquired factors, varicocele is considered to be the most common and correctable cause of male infertility [2, 3]. The incidence of varicocele is estimated to be 35–44% in males with primary infertility and 45–81% in males with secondary infertility [4]. The etiology of varicocele is multifactorial, and its pathogenesis is unclear [5]; however, studies have shown that reactive oxygen species (ROS) and the resultant oxidative stress (OS) play a central role in the pathogenesis of varicocele-related male fertility and are the key pathophysiological factors of male fertility caused by varicocele [6]. Due to the presence of varicocele, problems such as elevated testicular temperature, hypoxia, and OS will ultimately lead to testicular damage and abnormal sperm quality [7–10].

Varicocelectomy repairs and reduces ROS caused by varicocele, improves sperm DNA integrity and sperm quality, and is a common treatment for varicocele-related male infertility [11–13]. A meta-analysis has confirmed that sperm DNA integrity in patients with clinical varicocele is significantly improved following varicocelectomy [14]. The meta-analysis also found that adjuvant medical therapy after varicocelectomy improved sperm quality and sperm DNA integrity more than no medical treatment after varicocelectomy [15]. Vitamins are mainly used as antioxidants in the treatment of varicocele in infertile men; the application of antioxidants such as vitamin C and vitamin E after varicocelectomy has a positive effect on semen parameters in patients with varicocele-related male infertility, and even the application of vitamins alone can improve semen quality in some patients with varicocele-related male infertility without surgical treatment [16–18]. However, new evidence suggests that antioxidant supplementation on operated or non-operated does not improve semen parameters and pregnancy rates with varicocele-related male infertility without prior screening for OS [19].

However, ROS generated by OS is undoubtedly an important pathogenic factor in the entire varicocele process, affecting spermatogenesis through multiple pathways and mechanisms [20]. Especially for infertility caused by moderate-to-severe varicocele, whether it is necessary to use antioxidants, how potent antioxidants should be used, postoperative application, or non-surgical independent application still needs to be explored. Alpha-lipoic acid (ALA), chemically named 1,2-dithiolane-3-pentanoic acid, is an 8-carbon, cyclic disulfide antioxidant with the formula C_8_H_14_O_2_S_2_ and molecular weight 206.33 g/mol, which represents a promising antioxidant in the field of female and male infertility that inhibits oxygen-free radicals and rejuvenates oxidized antioxidants (i.e., glutathione and vitamins C and E) [21], as well as promotes the function of enzymes with antioxidant function. Animal experimental studies have shown that ALA can reduce the negative side effects of elevated testicular temperature and reduce OS in varicose rats [22]. It has also been clinically confirmed that ALA or ALA compound antioxidants can improve sperm motility and reduce sperm DNA damage in patients with varicocele after surgery [23, 24]. However, what role does ALA play in varicocele-related male infertility, and can it improve sperm quality more than traditional antioxidants? This is also a problem we think about and try to solve to help us formulate a more optimized treatment plan for varicocele-related male infertility.

### Objectives {7}

The general objective is to determine the efficacy and significance of ALA in varicocele-related male infertility through a randomized controlled clinical trial.

The specific objective is to determine the effect of different intensities of antioxidant conservative and surgical treatment of varicocele on sperm quality of varicocele-related male infertility patients by comparing surgical and non-surgical supplementation of ALA or vitamin E.

### Trial design {8}

**Fig. 1:**
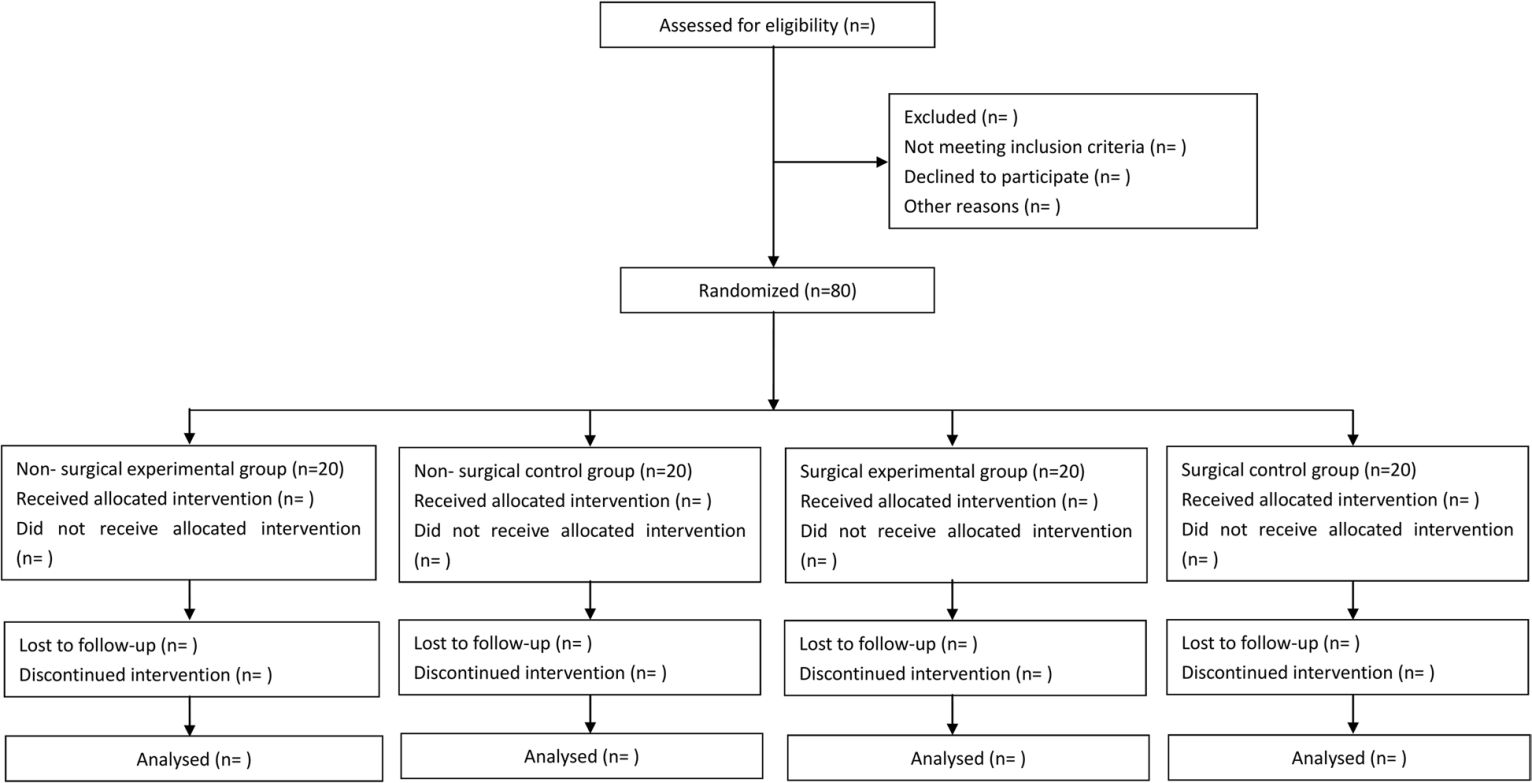
Flow chart describing the study protocol

This is a single-center, randomized, controlled, non-commercial clinical superiority trial evaluating the effectiveness of alpha-lipoic acid on sperm quality in patients with varicocele-related male infertility. The eligible participants will be allocated randomly into the non-surgical observation group, non-surgical control group, surgical observation group, or surgical control group in a ratio of 1:1:1:1. The four groups will be intervened for 3 months and followed up for 6 months. The flow chart is shown in Fig 1.

## Methods: participants, interventions, and outcomes

### Study setting {9}

The trial will be conducted in Guilin People’s Hospital, Guilin, China.

### Eligibility criteria {10}

The inclusion criteria are as follows:

- Infertile male aged 23–50 years
- Abnormal semen quality
- Spouse with normal fertility or suffering from infertility-related diseases, but may be cured
- Primary varicocele, physical examination to diagnose varicocele grade II (palpable in the standing position) or grade III (visible without palpation), color Doppler ultrasound measurement of the spermatic vein diameter at rest (DR) ≥ 2.8mm, time of reflux (TR) ≥ 4 s during the Valsalva maneuver.

The exclusion criteria are as follows:

- Pituitary or testicular organic diseases
- Azoospermia or chromosomal abnormalities
- History of vas deferens surgery or obstruction of the vas deferens cannot be ruled out
- Reproductive system infection
- Severe erectile dysfunction or ejaculation disorder
- Secondary varicocele
- Patients who have undergone varicocelectomy
- History of cancer, diabetes, or serious heart, liver, kidney, and hematopoietic diseases
- Severe mental illness
- Participates in other clinical trials or uses drugs that improve sperm quality or affect varicocele within the past 3 months
- Participants who have been using antioxidants or other nutritional supplements for a long time or within the past 3 months

### Who will take informed consent? {26a}

At baseline, the primary investigator will explain to patients the content and process of participating in the trial and obtain written informed consent upon enrollment.

### Additional consent provisions for collection and use of participant data and biological specimens {26b}

This trial involves collecting biological blood and semen samples. In the informed consent form, participants will be informed and asked if they consent to the use of their biological samples and data in future research.

### Interventions

#### Explanation for the choice of comparators {6b}

Vitamin E is an antioxidant commonly used in male infertility and a widely used adjuvant drug for varicocele and after varicocelectomy.

#### Intervention description {11a}

In the four groups, the non-surgical observation group will take a lipoic acid capsule 0.6 g, once a day; the non-surgical control group will take a vitamin E soft capsule 100 mg, twice a day; the surgical observation group will take a lipoic acid capsule 0.6 g, once a day; the surgical control group will take an oral vitamin E soft capsule 100 mg, twice a day. The course of treatment will be 3 months.

#### Criteria for discontinuing or modifying allocated interventions {11b}

In case of significant adverse drug reactions, the intervention will be stopped and reported to the Medical Ethics Committee of Guilin People’s Hospital. If the participant asks to stop the intervention, the intervention will stop.

#### Strategies to improve adherence to interventions {11c}

Monthly follow-up and record adverse drug reactions. Research participants can contact the investigators by phone or WeChat at any time, feed back drug use and treatment, and obtain guidance or suggestions.

#### Relevant concomitant care permitted or prohibited during the trial {11d}

Supplementation with vitamin E or ALA will not require alteration to usual care pathways.

#### Provisions for post-trial care {30}

Although known relevant studies suggest that the risks associated with this trial are small, the patients will be followed for 6 months after intervention and will be provided with adjunctive and post-trial care according to standard medical practice irrespective, regardless of the trial itself.

### Outcomes {12}

The primary outcome is the change of semen parameters (mainly progressive motility sperm) compared between the study groups at baseline and after 3 months of oral medication. Semen samples will be collected in sterile containers by masturbation after 2–7 days of abstinence. A semen analysis will be performed within 60 min after ejaculation and liquefaction using semen quality analyzer, and the semen volume, PH, sperm concentration, total motion, progressive motility (PR), and normal morphology will be measured according to the WHO laboratory manual for the examination and processing of human semen (5th edition) [25].

As secondary outcomes, the following will be measured at baseline and after 3 months of treatment: (1) Sex hormones, including follicle-stimulating hormone (FSH), luteinizing hormone (LH), estradiol (E2), and testosterone (T), will be determined by electrochemiluminescence using fasting blood. (2) Testicular volume will be calculated using Lambert’s formula (length × height × width × 0.71) [26]. (3) Sperm DNA fragment index (DFI) will be detected by flow cytometry-assisted sperm chromatin structure analysis (SCSA), seminal plasma malondialdehyde (MDA) levels will be detected by means of the tiobarbituric acid method, and seminal plasma total antioxidant capacity (TAC) will be detected by ferric-reducing antioxidant power (FRAP) assay method. (4) The pregnancy rate will be assessed during the 6-month follow-up after the completion of the trial.

### Participant timeline {13}

**Fig. 2:**
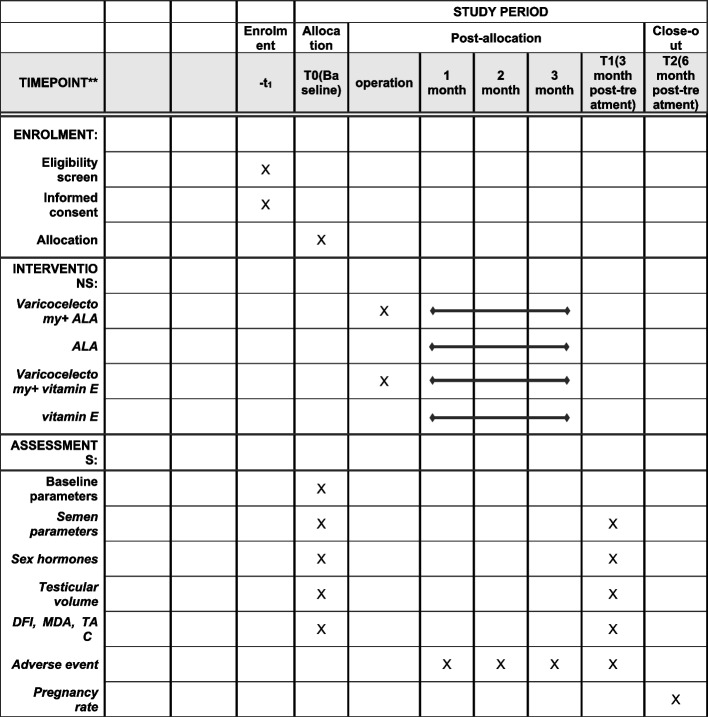
Data collection points

The participant timeline is presented in Fig. 2.

### Sample size {14}

Sample size estimation uses a completely random design to compare multiple sample means. The target sample size was estimated on PR sperm (as the primary outcome of this trial) using the PASS 2021 version 21.0.3 (NCSS, LLC, Kaysville, UT, USA). The number of samples considering the type I error *α* = 0.05 and the type II error *β* = 0.20 with 80% test power. According to a previous study [23], and a retrospective analysis of our data center in the past 2 years, changes in PR sperm mean value in the surgical observation group was 12.05, and the standard deviation was 3.96; changes in PR sperm mean value in the surgical control group was 6.56, and the standard deviation was 8.90; changes of PR sperm mean value in the non-surgical observation group was 4.15, and the standard deviation was 8.68; changes of PR sperm mean value in the non-surgical control group was 1.42, and the standard deviation was 7.60. After calculation, the sample size required for each group was 16 people. To reduce error, we slightly expanded the target sample size and estimated a dropout rate of 20%. The sample size will be practically 20 patients per group, and we will consider a total of 80.

### Recruitment {15}

Patients will be recruited from the outpatient department of Andrology and Urology of Guilin People’s Hospital.

### Assignment of interventions: allocation

#### Sequence generation {16a}

The allocation sequence will be generated by computer through the block randomization method, and the block length will be multiple and remain blind and will not be open to the subjects, researchers, and other relevant personnel participating in the trial. Qualified participants will be randomly divided into four groups at a ratio of 1:1:1:1.

#### Concealment mechanism {16b}

Allocation will be concealed in sequentially numbered, opaque, sealed envelopes containing the randomization assignments. After each participant meets the eligibility criteria and signs informed consent, the appropriate numbered, opaque, sealed envelope will be opened.

#### Implementation {16c}

The generation and allocation of sequences will be conducted by a designated researcher who is not involved in the data collection or analysis.

### Assignment of interventions: blinding

#### Who will be blinded {17a}

The grouping involves surgery, and non-surgery makes it impossible to blind the trial participants. However, the data collection staff, outcome assessors, and data analysts will be blinded. The varicocelectomy will be performed by the same surgeon and he will be blinded.

#### Procedure for unblinding if needed {17b}

Not applicable.

### Data collection and management

#### Plans for assessment and collection of outcomes {18a}

Data will be collected at three time points: (T0) pre-treatment (baseline) records—baseline parameters, sperm quality, sex hormones, testicular volume, sperm DFI, seminal plasma MDA, and TAC; (T2) 3 months post-treatment—sperm quality, sex hormones, testicular volume, DFI, seminal plasma MDA and TAC, and adverse event; and (T3) 6 months post-treatment—pregnancy rate.

#### Plans to promote participant retention and complete follow-up {18b}

The trial participant will be followed up by telephone or WeChat during treatment and 6 months after treatment.

### Data management {19}

All data will be kept confidential. Data will be stored electronically with encryption, allowing only authoring investigators to enter data. A participant identifier code for the data will be used so that data will not have the participant’s name associated with it. A separate key file connecting the participant name and participant identifier code will be created and stored in a secured location where only the principal investigator has direct access to the list. Participant identifier codes will be used for all data entry and data analyses.

### Confidentiality {27}

All manual data related to the trial will be safely stored in the restricted access location. Participant identification information, written informed consent, and other relevant sensitive data documents in the trial will not be exposed to anyone outside the research team.

### Plans for collection, laboratory evaluation, and storage of biological specimens for genetic or molecular analysis in this trial/future use {33}

Blood and semen samples will be collected and tested by the laboratory department of Guilin People’s Hospital in accordance with the standard testing procedures, and biological samples will not be preserved. No genetic studies are currently planned.

## Statistical methods

### Statistical methods for primary and secondary outcomes {20a}

Data analysis will be performed using IBM SPSS Statistics for Windows, version 24 (IBM Corp., Armonk, NY, USA). Compliance of variables with normal distribution will be analyzed by the Shapiro-Wilk test and homogeneity of variance by Levene’s test. Continuous variables will be expressed as mean with standard deviation (mean ± SD) or median (25 to 75%). Categorical variables will be expressed as numbers with percentages. Continuous data that are normally distributed and homogeneous in variance will be analyzed with one-way analysis of variance, and if the result is statistically significant, multiple comparisons will be performed with Bonferroni correction. Continuous data that do not obey normal distribution or have unequal variance will be analyzed with the Kruskal-Wallis test, if the result is statistically significant, then the Wilcoxon signed-rank test, with a Holm-Bonferroni method correction for multiple comparisons. Within groups before and after the intervention will be analyzed by paired-samples *t*-test for normally distributed variables and Wilcoxon signed-rank test for non-normally distributed variables. Categorical data will be analyzed using Pearson’s chi-square test or Fisher’s exact test. The level of significance will be set to *p* < 0.05.

### Interim analyses {21b}

No interim analyses will be performed.

### Methods for additional analyses (e.g., subgroup analyses) {20b}

No subgroup analyses are planned.

### Methods in analysis to handle protocol non-adherence and any statistical methods to handle missing data {20c}

When analyzing the results, we will conduct an intention-to-treat analysis for missing data. The last record data will be carried over to the end, and if a large number of missing data are found in further statistical analysis, multiple interpolation will be considered.

### Plans to give access to the full protocol, participant-level data, and statistical code {31c}

Statistical codes are available upon request.

### Oversight and monitoring

#### Composition of the coordinating center and trial steering committee {5d}

The Ethics Committee of Guilin People’s Hospital will supervise all the study stages. They will oversee the conduct of the trial at any unexpected time and will issue recommendations for early termination, modifications, or continuation of the trial, if necessary.

#### Composition of the data monitoring committee, its role, and reporting structure {21a}

This single-center study did not require a data monitoring committee.

#### Adverse event reporting and harms {22}

Any adverse events and other unexpected accidents during the study period will be recorded and reported to the Ethics Committee of Guilin People’s Hospital for decision-making.

#### Frequency and plans for auditing trial conduct {23}

The Ethics Committee will be responsible for monitoring the trial. Audits on accuracy may be carried out at any time and at least twice.

#### Plans for communicating important protocol amendments to relevant parties (e.g., trial participants, ethical committees) {25}

Protocol amendments will be approved by the Trial Steering Committee, and the ChiCTR.org.cn listing will be updated periodically for enrollment or major protocol changes.

#### Dissemination plans {31a}

The final study results will be published in international peer-reviewed journals focusing on the investigatory field in question.

## Discussion

The plasma membrane of sperm contains rich polyunsaturated fatty acids (PUFAs), which are extremely vulnerable to attack by ROS and eventually lead to lipid peroxidation and the formation of MDA. The level of seminal plasma MDA can reflect the production of oxygen free radicals in seminal plasma and the degree of causing lipid peroxidation, while TAC reflects the reduction potential in seminal plasma. Seminal plasma MDA and TAC are important indicators for the diagnosis and treatment of male infertility, have a clear correlation with the sperm quality of male infertility, and affect sperm quality under the regulation of antioxidant treatment [27–29]. Many uncontrolled oxidative stress factors, such as inflammatory reactions, bacteria, or viruses, may have an impact on male testes or reproduction [27, 30, 31], and thus, there seems to be a wide demand for antioxidant supplements in male reproduction. However, reducing stress may also be related to excessive supplement of antioxidants, and may aggravate ROS production, thus affecting sperm quality [32]. This requires us to examine the necessity of antioxidant supplementation.

Some animal experiments have shown that ALA supplementation can alleviate ROS, reduce testicular tissue damage, and improve testicular spermatogenesis and steroidogenesis disorders [33–36]. A small number of clinical studies have also found that ALA supplementation can improve the quality of semen parameters in male infertility [37, 38], and there are also fewer studies on the application of ALA in varicocele-related male infertility [23, 24]. Previous studies have mostly focused on surgical treatment of varicocele-related male infertility or postoperative supplementation with antioxidants, while this clinical trial intends to study both surgical and non-surgical treatment of varicocele-related male infertility patients and compare the effects of two antioxidants (ALA vs. vitamin E) on semen parameters, seminal plasma MDA and TAC before and after treatment, so as to reveal whether antioxidant use is necessary for varicocele-related male infertility, how potent should be used, non-surgical application, or postoperative application. Since our study involved surgical and non-surgical treatment options, some non-surgical patients are likely to choose further treatment options (varicocelectomy) after finishing the trial, which is also what we may recommend and according to the patient’s wishes. We may continue to follow up these patients who underwent surgery after antioxidant treatment.

## Trial status

The trial version is 1.0 and the protocol was approved on December 29, 2021. Recruitment will be started in June 2022 and the anticipated completion is December 2023.

## Data Availability

During the trial, all data will not be available to the public, but only to the research team.

## Abbreviations

ALA: Alpha-lipoic
ROS: Reactive oxygen species
OS: Oxidative stress
DR: Diameter at rest
TR: Time of reflux
PR: Progressive motility
NP: Non-progressive motility
FSH: Follicle-stimulating hormone
LH: Luteinizing hormone
E2: Estradiol
T: Testosterone
DFI: DNA fragmentation index
MDA: Malondialdehyde
TAC: Total antioxidant capacity
PUFAs: Polyunsaturated fatty acids
